# Prevalence and Antimicrobial Resistance of Virulent *Listeria monocytogenes* and *Cronobacter sakazakii* in Dairy Cattle, the Environment, and Dried Milk with the In Vitro Application of Natural Alternative Control

**DOI:** 10.3390/antibiotics11081087

**Published:** 2022-08-10

**Authors:** Basma Badawy, Mayada Gwida, Asmaa Sadat, Marwa EL-Toukhy, Mohamed Sayed-Ahmed, Nawazish Alam, Sarfaraz Ahmad, MD Sajid Ali, Mahmoud Elafify

**Affiliations:** 1 Department of Hygiene and Zoonoses, Faculty of Veterinary Medicine, Mansoura University, Mansoura 35516, Egypt; 2 Department of Bacteriology, Mycology, and Immunology, Faculty of Veterinary Medicine, Mansoura University, Mansoura 35516, Egypt; 3 Department of Food Hygiene and Control, Faculty of Veterinary Medicine, Mansoura University, Mansoura 35516, Egypt; 4 Department of Pharmacy Practice, College of Pharmacy, Jazan University, Jazan 82722, Saudi Arabia; 5 Department of Pharmaceutics, College of Pharmacy, Jazan University, Jazan 82722, Saudi Arabia

**Keywords:** *L. monocytogenes*, *C. sakazakii*, antimicrobial resistance, essential oils, alternative

## Abstract

This study aims to detect the prevalence and antimicrobial resistance of *Listeria monocytogenes* and *Cronobacter sakazakii* in three dairy households and dried milk from different suppliers, and evaluate the antimicrobial effect of rose water, rose, and orange essential oils. In total, 360 samples were collected from cattle, the environment, and dried milk (*n* = 30). Antimicrobial activity was evaluated with twofold microtube dilution and the time-kill method. *L. monocytogenes* was identified in all households (13.3%) with a prevalence in the range of 5.8–17.5%, while *C. sakazakii* was identified in one household (5.3%). The former and latter pathogens were highly isolated from the feces at 20% and 2.5% and bedding at 12.5% and 1.6%, respectively. *L. monocytogenes* was isolated only from milk at 7.5%, but *C. sakazakii* was not detected in either milk or dried milk. *L. monocytogenes* strains were screened for virulence genes (*iap, hylA,* and *actA*). All strains were positive for the *iap* gene, while for *hlyA* and *actA*, the percentages were (35.4% 16.6%, respectively). *L. monocytogenes* strains showed high resistance against sulfamethoxazole–trimethoprim (100%), followed by gentamicin, penicillin, and imipenem (95.8%, 95.8%, and 91.6%, respectively). All *C. sakazakii* strains were susceptible to all tested antibiotics. The bactericidal activity of orange oil was the strongest, appeared after 1 h for both pathogens, followed by rose oil and then rose water.

## 1. Introduction

Food-producing animals and food products of animal origin are the main reservoirs and vehicles of most zoonotic foodborne pathogens such as *Listeria monocytogenes* [1,2], or contributed in the infection cycle of *Cronobacter sakazakii* [3]. *L. monocytogenes* is an intracellular Gram-positive bacterium that is widely distributed in different natural environments, such as agricultural, aquacultural, and food-processing environments [4]. Infections by *L. monocytogenes* in humans and animals are characterized by eye infections, keratitis, encephalitis, septicemia, uterine infections resulting in abortions and still births, and subclinical mastitis [2]. *L. monocytogenes* pathogenicity is attached to its endurance to a variety of environmental stressors, such as osmotic, thermal, and chilling stressors. Foods are the main vehicle for *L. monocytogenes,* which is estimated to be the third major microorganism that can be transferred via food [1].

The *Cronobacter* genus, known before as *Enterobacter sakazakii,* is an abundant foodborne microorganism related to the Enterobacteriaceae family [5,6]. The genus contains several species, such as *C. sakazakii, C. malonaticus, C. dubliniensis, C. condimenti, C. muytjensii, C. turicensis,* and *C. universalis* [5]. *C. sakazakii* is a yellow-pigmented Gram-negative foodborne bacterium that was identified as an infrequently isolated opportunistic pathogen, and is found in a wide range of environmental sources, food, and spices [7]. The bacterium has received considerable attention, as it was seriously implicated in cases of sepsis, necrotizing enterocolitis, and meningitis, especially in low-birth-weight infants [8]. 

Currently, there are complex global public hazards owing to the existence of antimicrobial resistance (AMR), which mandates the application of new antimicrobial agents in order to combat pathogenic microorganisms [9]. Unfortunately, there is no legislation in Egypt regulating the use of antibiotics [10]. Antimicrobials such as tetracycline, quinolones, and beta lactams are still used in Egypt as growth promotors and feed additives in animal feedstuff by veterinarians or paravets for the treatment and prevention of zoonotic diseases [11]; subsequently, AMR is developed. In agreement with the One Health concept established by the WHO in 2017 to establish the fact that animal and human health is inter-related and connected to the wellbeing of the environments in which they coexist, various member states, including Ghana, were tasked to develop and implement an antibiotic resistance action plan. Consequently, a 5-year National Action Plan (NAP) on AMR (2017–2022) was officially launched in Ghana in April 2018, with two of five strategic objectives relating to the regular surveillance of AMR and the optimization of the administration of antimicrobial drugs in human medicine, plant production, and animal health. Lately, multidrug-resistant *L. monocytogenes* has been frequently isolated from food and the environment, and infrequently from humans. Hence, there is a need to monitor the antibiotic resistance patterns of *L. monocytogenes* and to reduce health problems associated with its infection [12].

Natural botanical materials and different crude extracts of spices, herbs, and essential oils (EOs) are naturally active elements that have health benefits and protective effects against diseases and foodborne pathogens [13]. For instance, the EOs of damask rose and orange oil are considered to be promising natural compounds that have antioxidant, antifungal, and antibacterial activity [14,15]. In spite of the verified effectiveness of these chemical preservatives in the inhibition of food poisoning and outbreak control, their frequent usage leads to chemical residue buildup in food and subsequently in the food chain, an increase in microbial resistance to such used chemical agents, and adverse side effects at the public health level [16]. The extracts of these plants are natural supplies of simply degradable and safe antimicrobial agents [16]. The bactericidal action shown by plant extracts against foodborne bacteria has been confirmed by an adequate number of scientists [17].

This context led us to study the potential existence, prevalence, and AMR of pathogenic virulent *L. monocytogenes* and *C. sakazakii* in household-reared dairy cattle, their environment, and dried milk collected from local markets. A household sector is an important dairy sector that represents a high risk for humans that consume such milk or milk products. The antimicrobial activity of some natural compounds that can be used as feed additives, such as rose water, rose, and orange oil, for both animals and farmers was also evaluated against the standard strains of the two pathogens under study. This study detects the existence of both virulent and multidrug-resistant *L. monocytogenes* and *C. sakazakii* in dairy animals and their environment, which negatively impact animal health, the dairy industry, and consumer health, and necessitate the application of strict hygienic measures. The study also confirms the antimicrobial activity of the tested products (particularly orange oil and rose oil) against both pathogens. Further studies are needed to confirm the effects of these natural products and oils in vivo, and detect the extent of its toxicity. They could also be used as part of feed additives for both animals and humans to eliminate antibiotic-resistant bacteria and become a part of food safety approaches. 

## 2. Results

### 2.1. Prevalence of L. monocytogenes and C. sakazakii in Cattle, the Environment, and Dried Milk

**Figure 1:**
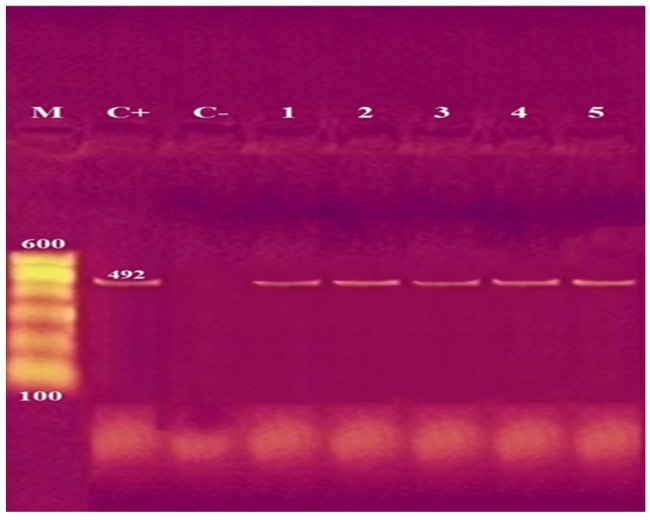
Agarose gel electrophoresis of PCR of a specific oligonucleotide sequence as part of *cgcA* (genus-specific marker sequences) at 492 bp for detection of *C. sakazakii***.** Lane M: 100 bp ladder as molecular size DNA marker. Lane C+: Control positive *C. sakazakii*. Lane C–: Control negative. Lanes 1 to 5: positive *C. sakazakii* strains.

Out of 360 samples, 48 isolates were positive for L. monocytogenes (13.3%; Household I, 17.5%; Household II, 16.6%; Household III, 5.8%), and five isolates were positive for *C. sakazakii* (5.3%; detected only in Household I, 4.1% (Figure 1). 

The prevalence of *L. monocytogenes* was in the range of 5.8–17.5% in the three examined households. The prevalence of *L. monocytogenes* in animals (13.7%) was similar to the environmental prevalence (12.5%). Fecal specimens had the highest isolation rate for *L. monocytogenes* and *C. sakazakii* (20% and 2.5%, respectively), followed by straw bedding materials at 12.5% and 1.6%, respectively. *L. monocytogenes* was isolated only from raw milk at 7.5%, but not isolated from dried milk samples. In contrast, *C. sakazakii* was not detected in either raw milk or dried milk samples (Table 1). All data were statistically significant at a *p*-value of less than 0.05. Dried milk had no bacterial contamination by either *L. monocytogenes* or *C. sakazakii.*

### 2.2. Antibiogram of Isolated Strains of L. monocytogenes and C. sakazakii

The AMR of *L. monocytogenes* strains was high in SXT (100%), followed by gentamicin, penicillin, and imipenem (95.8%, 95.8%, and 91.6%, respectively). By contrast, the resistance of *L. monocytogenes* strains to levofloxacin and vancomycin was low (16.6% and 2.1%, respectively). On the other hand, all *C. sakazakii* strains showed susceptibility to all tested antibiotics (Table 2).

### 2.3. Prevalence and Distribution of L. monocytogenes Virulence Genes with Their Antimicrobial Resistance Profile and Multiple Antibiotic Resistance (MAR) Index

**Figure 2:**
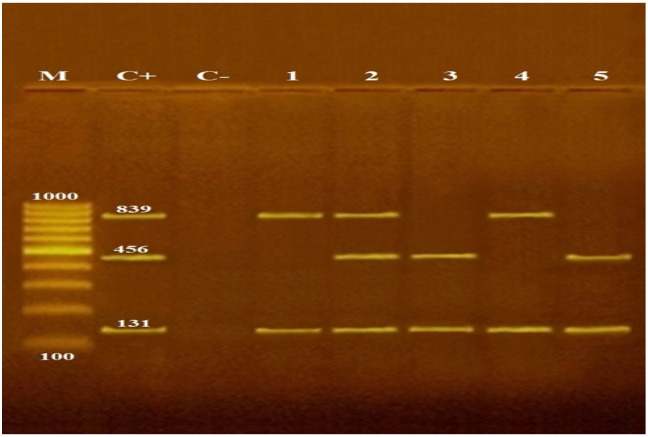
Agarose gel electrophoresis of multiplex PCR of *iap* (131 bp), *hylA* (456 bp) and *actA* (839 bp) virulence genes for the characterization of *L. monocytogenes*. Lane M 100 bp ladder as molecular size DNA marker. Lane C+: control positive *L. monocytogenes* for *iap*, *hylA* and *actA* genes. Lane C–: control negative. Lanes 1 and 4: positive *L. monocytogenes* strains for *iap* and *actA* genes. Lanes 2, 3, and 5: positive *L. monocytogenes* strains for *iap* and *hylA* genes. Lane 2: positive *L. monocytogenes* strain for *iap*, *hylA*, and *actA* genes.

All the identified *L. monocytogenes* strains were screened by multiplex PCR for the characterization of virulence genes (*iap*, *hylA*, and *actA*), and were all significantly positive (100%) for the presence of the invasion-associated protein expressed by the *iap* gene from the three examined households at the *p*-value (≤0.001, ≤0.001, and 0.011, respectively). On the other hand, listeriolysin and actin assembly expressed by the *hlyA* and *actA* genes were detected significantly in 19 and 7 isolates (39.6% and 14.6%, respectively). The recovery rate of the *hylA* gene was higher in Household II (45%) than that in Households I (38%) and III (28.5%). On other hand, the recovery rate of *actA* gene was higher in Household III than that in Households II and I (28.5%, 15%, and 9.5%, respectively). There was a concurrent recognition of the three *L. monocytogenes* virulence genes (*iap*, *hlyA*, and *actA*) in four *L. monocytogenes* isolates (two isolates from Household I from milk and bedding with MAR index values of 0.88 and 0.77, respectively, and two isolates from Household II from milk and bedding with MAR index values of 0.66, and 0.77, respectively). Of *L. monocytogenes* isolates, 4/48 (8.3%) had the three virulence genes and the highest MAR index among all tested strains (Table 3, Figure 2). Two of the three virulence genes (*iap*, *hlyA*, or *actA*) were also detected in 18 out of 48 isolates (37.5%) in which 6, 8, and 4 isolates were detected from Households I, II, and III, respectively.

The MAR index was higher in Household I (0.644) than that in Household II (0.610), which was higher than that of Household III (0.364). All examined *L. monocytogenes* strains *n* = 48 (100%) had a MAR in at least 3 of the tested antibiotics (*n* = 9). Their MAR index ranged from 0.33 to 0.88. The highest MAR index of 0.88 was recorded in 1 isolate out of 48 (2.1%) in milk from Household I, which had the highest prevalence of virulent *L. monocytogenes* among the three examined households. Furthermore, a MAR index of 0.77 was detected in 12.5% (6/48) of the isolates; 5 out of these 6 isolates (83.3%) were detected from Household I. Meanwhile, a MAR index of 0.66 was detected in 29.2% (14/48) of the isolates, in which 4 and 10 isolates (28.5% and 71.4%) were detected from Households I and II, respectively.

### 2.4. Antibacterial Activity Assessment of the Selected Natural Products against Standard Bacterial Strain

**Figure 3:**
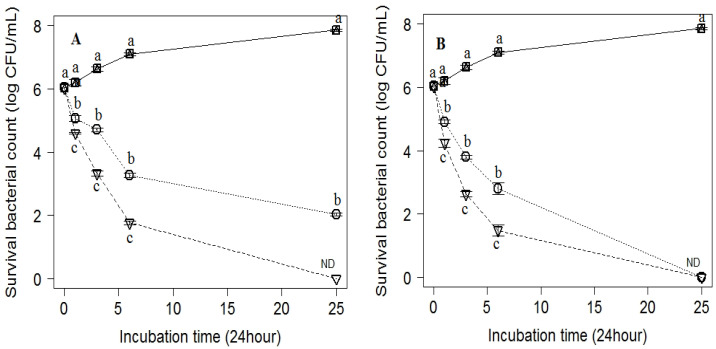

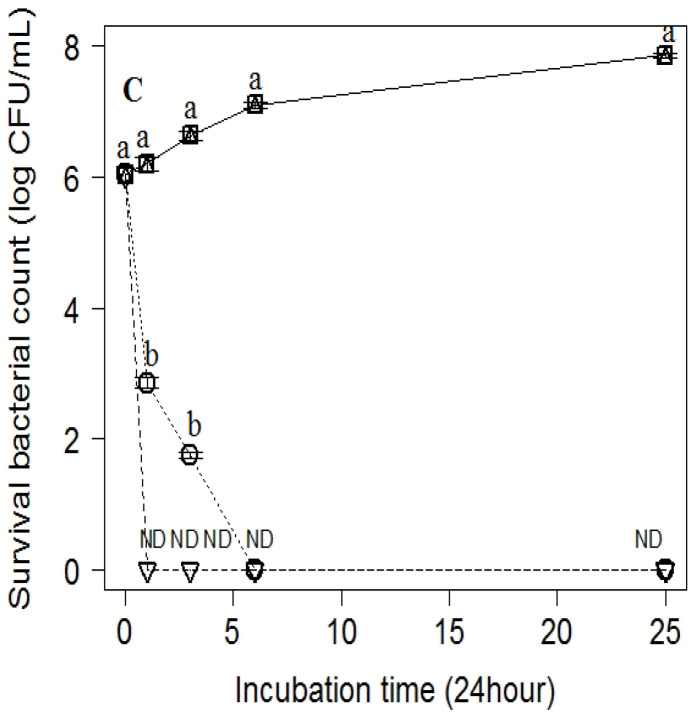
Inhibitory effect of (**A**) rose water, (**B**) rose oil, and (**C**) orange oil at different concentrations; (b) MIC (dotted line) and (c) 2 × MIC (dot-dashed line). (a) Control (solid line) on the viable counts of *L. monocytogenes* inoculated into MHB and incubated at different time points of 0, 1, 3, 6, and 24 h. Values are the mean ± standard deviation of three independent trials. ND means not detected.

According to our results, the solubility of the tested EOs in the culture media was very suitable when using Tween 80 as an emulsifier. Furthermore, 0.5% Tween 80 did not exhibit any antimicrobial effect in vitro. The antimicrobial effect of Tween 80 was excluded by observing the broth turbidity and viable bacterial count in nutrient agar in the control-positive well. Our findings are summarized in Table 4. Similar MIC and MMC values (10 and 7.8 mg/L) were expressed in *L. monocytogenes* for rose water and orange oil, respectively. For *C. sakazakii*, similar values for MIC and MMC were also recorded; 20 and 7.8 mg/L for rose water and orange oil, respectively. For rose oil, the MIC and MMC values for *L. monocytogenes* were 2.5 and 20 mg/L, respectively. Meanwhile, for *C. sakazakii,* the MMC value was 40 mg/L, which was twice the value of MIC (20 mg/L). Significant (*p* < 0.05) difference for the growth of the tested pathogens were reported in the time-kill profiles of rose water, rose, and orange oil against *L. monocytogenes* as Gram-positive bacteria and *C. sakazakii* as Gram-negative bacteria. By testing the rose water against *L. monocytogenes*, there was a significant reduction in viable bacterial count (≥3 × log 10 cfu/mL) at 2 × MIC over the first 3 and 6 h, and at MIC at 6 and 24 h, but after 24 h at 2 × MIC, the pathogen was no longer detected (Figure 3A). After the treatment of *L. monocytogenes* with rose oil, there was significant bactericidal activity and bacterial log reduction after 3 and 6 h at 2 × MIC, and at MIC after 6 h, but after 24 h, the pathogen was not detected in either MIC or 2 × MIC (Figure 3B). Orange oil exhibited bactericidal activity at an MIC concentration against *L. monocytogenes* at 1 and 3 h, and the bacteria were no longer detected at 6 and 24 h (Figure 3C). 

**Figure 4:**
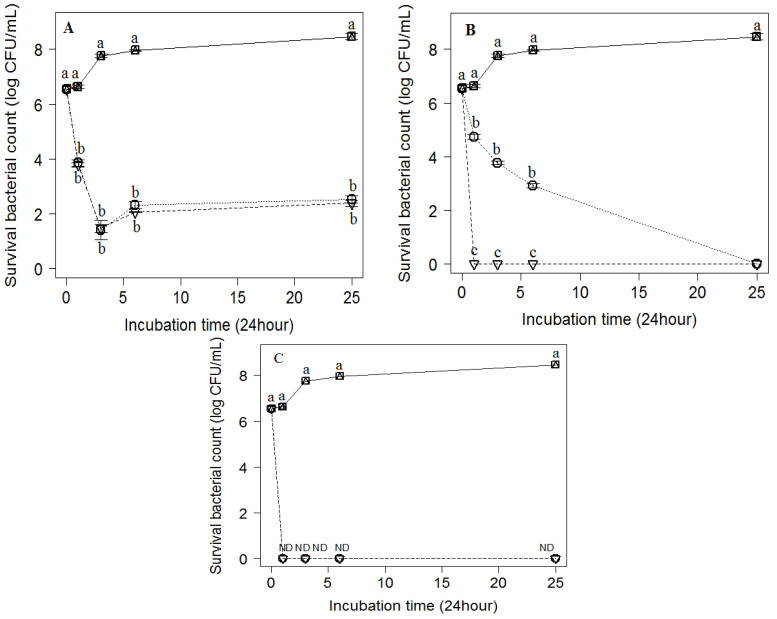
Inhibitory effect of (**A**) rose water, (**B**) rose oil, and (**C**) orange oil at different concentrations; (b) MIC (dotted line) and 2 × MIC (dot-dashed line). (a) Control (solid line) on the viable counts of *C. sakazakii* inoculated into MHB and incubated at different points of time (0, 1, 3, 6 and 24 h). Values are the mean ± standard deviation of three independent trials. ND, not detected.

On the other hand, strong bactericidal activity (around 6 × log 10 cfu/mL) was recorded for rose water against *C. sakazakii* at MIC and 2 ×MIC at the contact time of 3 h. This was followed by slight growth at 6 h, decreasing the bacterial log to 5.5× log 10 cfu/mL, followed by a gradual increase to 6 × log 10 cfu/mL after 24 h of contact time (Figure 4A). Rose oil exhibited a strong bactericidal effect against *C. sakazakii* from 1 till 6 h at MIC with gradual decrease in bacterial count till 24 h, when the pathogen was no longer detected. At the 2 ×MIC concentration of rose oil, there was complete inhibition of bacterial growth after the first hour of the application of rose oil (Figure 4B). Orange oil at MIC and 2 ×MIC exhibited noteworthy antibacterial activity against *C. sakazakii* from the first hour of treatment till the end of the experiment after 24 h (Figure 4C). The most effective natural product was orange oil, followed by rose oil and then rose water.

## 3. Discussion

The current study estimated *L. monocytogenes* and *C. sakazakii* prevalence in household-reared dairy cattle, their environment, and dried milk as a milk product, and detected the existence of virulent *L. monocytogenes* in raw and dried milk. The laboratory trials of the microbial reduction in the reference standard strains of the two microbes under study were performed by using natural products as an alternative therapy to the traditional chemical agents to reduce the growing risks of AMR. Regarding the presence of *L. monocytogenes* in feces, milk, and environmental samples collected from dairy, the findings of Kim and his colleagues [18] were consistent with ours, indicating that bovine feces, environmental dairy farm samples, and raw milk can hold a varied set of strains of *L. monocytogenes.* In Egypt, lower and higher prevalence rates of *L. monocytogenes* than ours were detected at 7.23% and 28.1% in dairy farms in studies conducted by Elsayed et al. and Mohammed and Abdel Aziz, and, respectively [2,19]. These differences in results could be the result of the difference in hygiene level of the farms, varying farming practices, and different weather conditions where the sampling and research study were performed. The animal prevalence of *L. monocytogenes* was higher than that of the environment. These findings did not match the findings of Mohammed and Abdel Aziz [19], who detected environmental prevalence (30%) to be higher than animal prevalence (26.30%). Elsayed et al. [2] also detected environmental prevalence (8.3%) to be higher than animal prevalence (6.8%). In the current study, *L. monocytogenes* was identified in 20% of the examined dairy cow fecal samples. A similar proportion of fecal shedding was seen in a longitudinal study throughout the course of a year, as reported by Bandelj et al. [4], who detected *L. monocytogenes* in 18.2% of pooled cow fecal samples with prevalence in the range of 3.7–40.7% among examined farms, while a higher detection rate (46.3%) was recorded from fecal samples of dairy cattle by Esteban et al. [20]. *L. monocytogenes* is widely present in cattle feces and serves as a significant reservoir of *L. monocytogenes,* as mentioned by Bandelj et al. [4], who attributed the difference in the percentage of *L. monocytogenes* in fecal samples between our study and others to the high day-to-day variation in *L. monocytogenes* shedding in cow feces. Both humans and animals are asymptomatic carriers of *L. monocytogenes,* and are able to excrete the pathogen in farm environments [21]. The circulation of *L. monocytogenes* into the surrounding environment from different contamination sources has an adverse influence on the dairy industry, and public health could be the main source of animal infection [22].

*L. monocytogenes* is more commonly detected in fecal samples than in milk samples, as described in our study and other studies agreeing with our results [2] (6.8% and 5.9%, respectively). Regarding the prevalence of *L. monocytogenes* in milk in Egypt, there were variations recorded by [1,23] by36.7 %, 6%. In contrast, the authors in [24] failed to identify *L. monocytogenes* from the examined raw milk samples. The existence of *L. monocytogenes* in raw cow milk might be from exogenous sources, due to contamination by fecal matter during the milking process, or, less commonly, by an intramammary way after general asymptomatic infection or mastitis [21] or through the ingestion of contaminated silage, water, other ecological items contaminated with *L. monocytogenes*, wildlife, and/or from contact with the fecal matter of other cows that are shedding this organism. This results in a variety of possible ways for *L. monocytogenes* to spread within dairy flocks, and thereby a high level of strain diversity [18]. Variation in *L. monocytogenes* prevalence in milk may be due to variation in the hygiene level of farms or in adopting all the hygienic measures of the milking process, from milking order starting from healthy to diseased cows till the disinfection of milking equipment and udders during and after the milking process. 

On the basis of our data, 15 (12.5%) out of 120 straw bedding samples were contaminated with *L. monocytogenes*. Various detection rates were reported: 55% by Mohammed et al. [25], and 5–35% by Castro et al. [22]. Listeria was detected in raw milk samples. In contrast to our findings, Rodas-Suzáre et al. [26] did not determine *Listeria* species in milk, but detected it in dry skimmed milk samples at 7.8% out of 550 isolates, of which 23 were recognized as *L. monocytogenes*. The authors attributed the presence of *Listeria* in the dry milk samples to postprocessing contamination, improper hygienic measures, or contamination during packaging. The distribution of virulence genes (*iap, hylA*, and *actA*) in the confirmed *L. monocytogenes* plays a significant role in its pathogenicity. Genes *iap* and *hlyA* are responsible for host cell invasion, while the *actA* gene is associated with cell-to-cell spread. Our finding agreed with those of previous studies conducted by Tahoun et al., and Şanlibaba et al. [1,2,3,4,5,6,7,8,9,10,11,12,13,14,15,16,17,18,19,20,21,22,23,24,25,26,27], in which virulence genes were determined in *L. monocytogenes* isolated from raw milk, animals, and environmental samples. Our findings in virulence gene detection are also supported by the findings of [23], who reported that virulence genes *hlyA*, *iap,* and *actA* were the most recognized, with prevalence rates of 70.6%, 70.6%, and 52.9%, respectively, and two of the three virulence genes (*hlyA*, *iap* or *actA*) were simultaneously detected in six isolates.

Regarding the circulation of *C. sakazakii* in the examined samples, there were different occurrence rates of *C. sakazakii* in bovine feces previously reported by Awadallah et al. [8] (4%), and Ogihara et al. [28] (37.5%). The results obtained by Ogihara et al. [28] suggested that bovine feces might be one of the potent natural habitats of *Cronobacter* species, as they were able to isolate this species from bovine feces, soil, and compost by 37.5%, 16.7%, 10.0%, respectively). However, other researchers recorded that food production animals are not part of the dissemination cycle of *Cronobacter* species [29,30]. *C. sakazakii* was not detected in dried milk samples in findings reported by El-Gamal et al. [31], and Awadallah et al. [8], who failed to isolate *C. sakazakii* from dried milk. *C. sakazakii* colonizes different environments due to its capability to adapt to several environmental stresses and its capacity to form a biofilm that facilities its survival in the food production chain [32].

Regarding the antibiogram, *L. monocytogenes* in the present study showed full resistance to the sulfonamide antibiotic group represented by SXT, followed by streptomycin, penicillin G, and imipenem (95.8%, 95.8%, 91.6%, respectively). Our results agree with those of another study [33], which detected the full resistance of *L. monocytogenes* strains isolated from raw milk, bulk tank milk, and soft chesses as milk products against penicillin and streptomycin, followed by vancomycin (81.5%), sulfamethazole/trimethoprim (70.4%), gentamicin (48.2%), and amikacin (40.7%). A high level of penicillin resistance was also recorded in [2]. In another study [34], all the isolates of the *Listeria* species were observed to be resistant against penicillin and imipenem (100%), followed by trimethoprim (75%). Another study [35] showed resistance to carbapenems, imipenem, and meropenem in 4% and 5% of strains collected from humans, animals, and food products in Russia, respectively. On other hand, 100% resistance to imipenem was recorded in [34]; however, gentamicin and meropenem appeared to be the most effective antibiotics, as all *Listeria* species isolates were susceptible to them. The authors in [36] found that *L. monocytogenes* strains isolated from fecal and fetal samples from slaughtered pregnant cows were 100% resistant to meropenem. There is a shortage of studies testing the imipenem antibiotic against *L. monocytogenes*. Vancomycin susceptibility (97.9%) was the highest among all tested antibiotics, as recorded in [2], which found that all *L. monocytogenes* strains (100) were susceptible to vancomycin. In contrast, all *L. monocytogenes* strains were resistant to vancomycin in [35]. Our findings reveal that 100% of the examined *L. monocytogenes* had MAR ranging from 0.33 to 0.88, which matches the result of Elsayed et al. [2]. All our examined *L. monocytogenes* strains were multidrug-resistant (resistant to three or more antibiotics), which matched the results in [33]. In the present findings, all *L. monocytogenes* strains had an MAR index of more than 0.20, which indicated that the strains isolated from the three dairy households came from highly contaminated sources in which there was antibiotic abuse, which represents a great potential risk regarding antimicrobial resistance.

Our result of the *C. sakazakii* antibiogram agreed with the findings in [32]. In a study conducted on 70 strains of *C. sakazakii* and *C. malonaticus* collected from powdered infant formula and processing environments, all the isolated strains were susceptible to most of the examined antibiotics: amikacin, ampicillin–sulbactam, aztreonam, cefepime, cefotaxime, ceftazidime, chloramphenicol, ciprofloxacin, colistin, gentamicin, imipenem, levofloxacin, meropenem, moxifloxacin, piperacillin, piperacillin–tazobactam, tetracycline, and trimethoprim–sulfamethoxazole. However, they were resistant to amoxicillin–clavulanate, ampicillin, and cefazolin [37]. In contrast, *C. sakazakii* showed multidrug resistance in study conducted by [38], and resistance to trimethoprim and/or neomycin [39]. The current study shows that the used natural products exhibited antibacterial activity against bacterial growth with varied effectiveness. According to our recorded findings, Tween 80 (0.5%) showed no antimicrobial activity, as previously recorded by Hamoud and his colleagues [17]. According to the CLSI (2018), the bactericidal effect of any given antimicrobial agent (including those of EOs) exists when it produces ≥3 × log 10 (99.9%) reduction in cfu/mL after 18–24 h of incubation in a broth under a given set of circumstances. The antibacterial activity of *Rose damascena* extracts (rose water and oil) was previously evaluated by Androutsopoulou et al. [40]. The authors demonstrated that rose oil and its aqueous extracts have sufficient broad-spectrum microbicidal activity, and these findings are in line with our data. A study by Shohayeb et al. [41] assessed the antimicrobial activity of *R. damascena* essential oils and petal extracts against both bacteria and fungi. The authors reported that rose oil and all tested rose fractions exhibited a significant microbicidal effect against several Gram-positive and Gram-negative bacteria. In contrast, Mostafa et al. [16] reported that rose oil was ineffective against 23 Gram-negative and -positive bacterial and fungal strains, including *L. monocytogenes* and *C. sakazakii*. The authors attributed their results to the source of the oil, variations in the number and and concentration of active compounds. The antibacterial outcome of rose oil was possibly due to its active ingredients, such as citronellol and geraniol, which are fast-acting compounds that can disable pathogens by disrupting cellular membrane integrity or function, and due to the presence of other substances, such as nerol, 2 phenylethanol, nonadecane, and heneicosane [40]. 

In the current study, orange oils expressed the highest inhibitory effect against both Gram-positive (*L. monocytogenes*) and Gram-negative (*C. sakazaki)* bacteria. This finding agreed with that by Settanni et al. [42], who reported that the EOs of citrus fruits could exhibit an inhibitory effect against a wide range of foodborne pathogens, including *L. monocytogenes* and *Salmonella enteritidis*. In another study, the antibacterial activity of hexanic extracts of orange oils was produced at a MIC value of 15 mg/mL against *L. monocytogenes* (ATCC 7644), while the essential oil of Moro Solarino orange peels showed less activity, with an MIC value of 92 mg/mL against the same bacterium [43].

The current study also reveals that *C. sakazakii* was more sensitive to the tested natural bactericidal agents than *L. monocytogenes* was. This finding agrees with that by Fraňková et al. [44], who evaluated the bactericidal activity of a variety of compounds extracted from different plants, 5 EOs, and an extract of propolis against *C. sakazakii* and *C. malonaticus*. In contrast, six different EOs from citrus fruits, including *Citrus sinensis* (orange oil), had previously had no antimicrobial effect on the growth of different *Cronobacter* species [32]. The bactericidal effect of orange oil can probably be attributed to the existence of certain components that are typically present in all citrus plants such as: limonene and pinene (α- and β-), and citral [44]. The authors studied the antimicrobial effect of limonene on *C. sakazakii* and *C. malonaticus,* and reported an MIC value of 0.3%, while α- and β-pinene elicited MIC > 0.5% on the same bacteria. Other researchers mentioned that citral as a component of citrus plants inhibited the growth of *C. sakazakii* through changes in cytoplasmic pH and ATP concentration [45].

## 4. Materials and Methods

### 4.1. Sampling

The present research study was carried out in three dairy cattle households located at El Mahalla El-Kubra, Gharbia Governorate, Egypt from August 2020 to February 2021. All examined households in this study had a history of health issue problems with a decrease in milk production. In total, 360 samples were collected from the animals and their environment, and dried milk samples (*n* = 30) were purchased randomly from local retail markets supplied by different producers. Fecal and milk samples (40 per household), and straw bedding materials (*n* = 40 per household) were collected as animal and environmental samples from the examined households. After the cleaning, washing, drying, and disinfection of the udders by ethyl alcohol, and discarding the fore milk, 10 ml of the milk was collected from each quarter, and the quarter samples were pooled per animal in one sample in a sterile tube. For the fecal specimens, the samples were directly collected from the animal rectum by using sterile gloves, while 100 g of the bedding was collected from five different locations in each cattle household and placed onto sterile plastic bags. The samples were collected on the basis of convenience and were transported immediately to the laboratory of the Hygiene and Zoonoses Department, Mansoura University, Egypt for further analysis. The owners consented to participating in the study and for the research plan to be conducted. 

### 4.2. Bacterial Isolation and Identification

Samples were evaluated for the existence of *L. monocytogenes* according to International Organization for Standardization (ISO) protocol 11290 (1996) as described by [46]. For primary enrichment, 25 g or 25 mL of sample was processed in 225 mL of Half Fraser Broth (Oxoid, Basingstoke, UK) and incubated for 24 h at 30 °C. After that, 0.1 mL of the pre-enrichment broth was mixed with 10 mL of Fraser Broth (Oxoid, Basingstoke, UK) and incubated for 48 h at 35 °C. Subsequently, a loopful from the cultured broth was cultured in Oxford Listeria Selective Agar (Oxoid, Basingstoke, UK) and incubated for 48 h at 35 °C. Several suspected colonies were selected and cultured on Trypton Soya Agar containing yeast extract (Oxoid, Basingstoke, UK), and incubated for 48 h at 35 °C. Suspected *Listeria* colonies were then checked biochemically and microscopically by Gram staining. The purified strains were subjected to a group of biochemical tests: oxidase, catalase, and sugar ‘fermentation tests with L-rhamnose, xylose, D-glucose, and mannitol, as performed in [2], and typical umbrella motility at 25 °C. The isolates that exhibited a positive reaction were subjected to the production of hemolysin through the usage of blood agar media (Oxoid, UK) complemented with 5% sheep blood. Lastly, the isolates were exposed to serological recognition using a *Listeria* latex agglutination kit (Oxoid, Basingstoke, Hampshire, England), a rapid test for the preliminary recognition of *Listeria* species in selective enrichment cultures. The Oxoid Listeria Test Kit proves the existence of *Listeria* species in a culture, and should be applied in combination with biochemical recognition for the full detection of *L. monocytogenes* [47].

*C. sakazakii* isolation was performed according to the protocol of ISO (ISO/TS 22964:2006): 25 g or 25 mL from milk, straw bedding, and dried milk was added to 225 mL of buffered peptone water (BPW) for pre-enrichment; for the fecal specimens, 1 g was pre-enriched in 9 mL BPW at 37 °C for 24 h. Afterwards, 100 µL of the pre-enriched broth was suspended in 10 mL of *Cronobacter* screening broth (CSB 38948, Sigma-Aldrich, USA, Michigan), incubated at 42 °C for 24 h, and tested for yellow coloration production. An aliquot from the enriched CSB was cultured on *Cronobacter* chromogenic agar (Sigma-Aldrich, St. Louis, MO, USA) and incubated at 44 °C for 24 h for the biochemical detection of *C. sakazakii* [5]. Strains showing standard characteristics were further tested with biochemical identification kits for confirmation using API 20E (BioMerieux, Durham, NC, USA). The identified strains of *L. monocytogenes* and *C. sakazakii* were stored in sterilized glycerol at –20 °C for further characterization. 

### 4.3. DNA Extraction

Bacterial DNA was extracted from the identified strains using a Gene JET Geneomic DNA Purification Kit (Fermentas) in accordance with the manufacturer’s instructions. The extracted DNA of *L. monocytogenes* was subjected to PCR using specific primers for virulence genes *iap, hlyA,* and *actA* by multiplex PCR, according to the method described by Kauer et al. [48]. The multiplex PCR was performed in 50 μL reaction volume. The PCR conditions were: at 95 °C, the initial denaturation of DNA was carried out for 2 min. After that, for 35 cycles each, denaturation at 95 °C for 15 s, annealing at 60 °C for 30 s, and extension at 72 °C for 1 min. Afterwards, there was a final extension at 72 °C for 10 min kept at 4 °C. Amplified DNA fragments were evaluated with 1.5% agarose gel electrophoresis in a 1xTBE buffer dyed with ethidium bromide, and taken and imaged on a UV transilluminator. The extracted DNA of *C. sakazakii* was further confirmed with the amplification of a specific oligonucleotide sequence as a part of *cgcA* (genus-specific marker sequences) in a total volume of 25 μL. Cycle conditions were as performed by Carter et al. [49]. The protocol was as follows: initial denaturation for 3 min at 94 °C, followed by 25 cycles of denaturation adjusted for 30 s at 94 °C, annealing for 30 s at 62 °C, extension for 60 s at 72 °C, and a final extension for 5 min at 72 °C. The amplified products were exposed to 1.5% agarose gel electrophoresis dyed with ethidium bromide, and were pictured and photographed under an ultraviolet transilluminator. *L. monocytogenes*-positive control (ATCC 19118) and *C. sakazakii*-positive control (ATCC 24135) were run alongside the tested isolates; these reference standard strains were obtained from the American Type culture collection (Manassas, VA, USA). All tested primer sequences and amplicon sizes are presented in Table 5. 

### 4.4. Antibiotic Resistance of L. monocytogenes and C. sakazakii Isolated from Dairy Cattle and the Environment

Antibiotic resistance was established with the agar disk diffusion method on Mueller–Hinton agar (Difco), as recommended by the Clinical and Laboratory Standards Institute [50]. Frequently applied antibiotics for humans and animals were selected to be tested against our isolated strains of *L. monocytogenes* and *C. sakazakii.* Nine antimicrobial discs (Oxoid) that related to six different antibiotic classes were used: imipenem (10 μg), penicillin G (10 U) related to β-lactams; erythromycin (15 μg) belonging to macrolides; amikacin (30 μg); gentamicin (10 μg); streptomycin (10 μg), related to aminoglycosides; vancomycin (30 μg) belonging to glycopeptides; sulfamethoxazole–trimethoprim (SXT; 5 μg), belonging to sulfonamides; and levofloxacin (5 μg), belonging to quinolones. The examined strains were assessed as susceptible, intermediate, or resistant in accordance with the CLSI guidelines for *Staphylococcus aureus* ATCC 25,923 and *Escherichia coli* ATCC 25,922 [50]. To ensure data compatibility, the experiment was repeated with positive and negative controls. The positive controls (quality control organism) were *L. monocytogenes* (ATCC 19118) and *C. sakazakii* (ATCC 24135). The negative control was 30 μL of sterile distilled water pipetted onto a blank disc (typically 6 mm in diameter). The data of antibiotic resistance were only presented when the quality control test findings were within satisfactory ranges. Strains exhibiting resistance to at least one antimicrobial drug in three or more antimicrobial categories were considered to be multidrug-resistant (MDR) strains [23]. The multiple antibiotic resistance (MAR) index was calculated by dividing the total amount of antimicrobial resistance for each isolate by the total number of tested antimicrobials, according to [2]. An MAR index value greater than 0.2 means that the isolates originated from a high-risk source of contamination where antibiotics are massively applied [2].

### 4.5. In Vitro Trials for Microbial Reduction in L. monocytogenes and C. sakazakii with Rose Water, Rose, and Orange EOs 

#### 4.5.1. Preparation of Bacterial Suspension

One or two pure colonies from each reference bacterial strains of *L. monocytogenes* (ATCC 19118) and *C. sakazakii* (ATCC 24135) gained from American Type culture collection (Manassas, VA, USA) were directly placed in 0.85% saline to obtain turbidity equal to 0.5 of the McFarland standard ≈ 1 × 10^8^ colony forming unit per ml (cfu/mL), and then diluted to obtain a final concentration of 10^6^ cfu/mL [17]. 

#### 4.5.2. Preparation of Plant Extract Products

Samples of the original solution (100%) of rose water, rose, and orange oils were obtained from Nefertari Company for Extracting Natural Herbs and Cosmetics (Cairo, Egypt). All plant extracts were sterilized by filtration with 0.45 µm Millipore filters. The selected EOs (rose and orange oil) were dissolved before testing with Tween 80 as an emulsifier (0.5% *v*/*v* to enhance oil solubility) to have a stock solution with the concentration of 40 mg/L for rose oil, as performed by [38], and 250 mg/L for orange oil, as conducted by Prabuseenivasan et al. [51]. The final concentration of Tween 80 in the experiment did not go above 0.5% (*v*/*v*). Rose water was diluted with sterilized distilled water to obtain a concentration of 40 mg/L, as performed by [40].

#### 4.5.3. Determination of Minimal Inhibitory Concentration (MIC) and Minimal Microbicidal Concentration (MMC)

The MICs and MMCs of the selected products against the tested organisms were established with the broth microdilution method as described by the Clinical and Laboratory Standards Institute guidelines (CLSI 2018) using 96-well microtiter plates with the purpose of evaluating and quantifying the antimicrobial activity of the tested natural products. Each well was filled with 100 μL of the consecutive dilution of rose water and rose oil (twofold serial dilutions) to obtain concentrations ranging from 40 mg/L in 1st first well to 0.078 mg/L in the 10th well, 250 mg/L for orange oil from in the 1st well to to 0.48 mg/L in the 10th well. Then, 100 µL of each bacterial culture broth 1 × 10^6^ CFU/mL in Muller Hinton Broth (MHB) was placed in each well. Lastly, the positive and negative controls were incorporated in each plate in the wells (11 and 12, respectively). The positive control containing only 195 μL of MHB had Tween 80 and 5 μL of bacterial culture broth; the negative control had 200 μL of MHB having Tween 80 with no bacterial inocula. The positive control was included in the experiment to exclude the antimicrobial effect of Tween 80. Meanwhile, the negative control was used to confirm the sterility condition of the experiment. After mixing, the plate was covered with a paraffin sheet to avoid overnight evaporation in the incubator, and incubate the plates were incubated for 18–20 h at 37 °C. MIC was determined as the lowest concentration that exhibited no bacterial growth or turbidity [41]. Furthermore, 3 μL was taken from the wells without visible growth, and inoculated into nutrient agar plates and incubated at 37 °C overnight. Three independent trials for each experiment were carried out.

### 4.6. Time-Kill Assay

The time-kill assay for the used natural products was performed following the procedure carried out by Hamoud et al. [17]. In brief, a loopful from the initial bacterial suspension of 1 × 10^6^ CFU/mL of the test organism was added and incubated at 37 °C with a concentration of the used natural products equal to MIC and twice MIC (2x MIC). Aliquots of 0.1 mL of the bacterial culture broth were taken at the different contact times of 0, 1, 3, 6, and 24 h, cultured aseptically into nutrient agar plates, and incubated for 24 h at 37 °C to determine the viable bacterial count in cfu/mL in the test medium. Three independent trials were performed for each dilution. As reported by the National Committee for Clinical Laboratory Standards (NCCLS), an antimicrobial agent is bactericidal when it produces 3 or more log 10 reduction (99.9%) cfu/mL after 18–24 h of incubation in a broth under a given set of circumstances. The antimicrobial agent definition is also used for EOs. The cfu/mL of the organisms was determined and recorded in an Excel sheet, and a graph of the log CFU/mL/ each natural product against the tested bacteria was plotted against time. 

### 4.7. Statistical Analysis

The prevalence of *L. monocytogenes* and *C. sakazakii* in household-reared cattle, their environment, and the prevalence and distribution of the virulence genes of *L. monocytogenes* were evaluated by using the Statistical Package of Social Science (SPSS) program for Windows (Standard version 26). Qualitative data were described using numbers and percentages. The correlation between different variables was analyzed by using the chi-squared and Fisher exact (used when expected cell count less than 5) tests. The threshold of significance was fixed at the 5% level. The result was considered to be significant when *p* ≤ 0.05. The smaller the obtained *p*-value was, the more significant the results were. The significance differences in the viable count of *L. monocytogenes* and *C. sakazakii* before and after the application of rose water, rose, and orange oil were assessed by *t*-test at *p* < 0.05 via R_ statistical software (v. 3.5.2, R foundation for statistical computing, Vienna, Austria)

## 5. Conclusions

In conclusion, the results of the current study provided information regarding the prevalence and AMR of pathogenic virulent strains of *L. monocytogenes* and *C. sakazakii* in the fecal specimens and milk samples of the dairy cattle of smallholders, and their surrounding environment and dried milk, which has a negative impact on animal health, the dairy industry, and consumer health, and necessitates the application of strict hygienic measures. The existence of multidrug-resistant *L. monocytogenes* in all examined samples, including milk samples, represents a great hazard to both animal and human health. The study also confirmed the antimicrobial activity of the tested products (particularly orange oil and rose oil) against *L. monocytogenes* and *C. sakazakii.* Hence, further studies are needed to confirm the effects of these natural products and oils in vivo, and detect the extent of its toxicity, using them as part of feed additives for both animals or humans to eliminate antibiotic-resistant bacteria and become part of food safety approaches. 

## Figures and Tables

**Table 1:** Prevalence of *L. monocytogenes* and *C. sakazakii* isolated from the environment of household-reared cattle.

**Animal House**	**Samples**	**Total Examined No**	***L. monocytogenes*** **-Positive No. (%)**	***C. sakazakii*** **-Positive No. (%)**	***p*-Value**
Household I	Animal samples				
	Fecal samples	40	9 (22.5)	3 (7.5)	0.06
	Milk	40	5 (12.5)	0 (0)	0.02
	Total	80	14 (17.5)	3 (3.7)	0.004
	Environment				
	Straw bedding	40	7 (17.5)	2 (5)	0.076
	Total	120	21 (17.5)	5 (4.1)	0.001
Household II	Animal samples				
	Fecal samples	40	11 (27.5)	0 (0)	≤0.001
	Milk	40	4 (10)	0 (0)	0.04
	Total	80	15 (18.7)	0 (0)	≤0.001
	Environment			0 (0)	
	Straw bedding	40	5 (12.5)	0 (0)	0.02
	Total	120	20 (16.6)	0 (0)	≤0.001
Household III	Animal samples				
	Fecal samples	40	4 (10)	0 (0)	0.04
	Milk	40	0 (0)	0 (0)	-
	Total	80	4 (5)	0 (0)	0.043
	Environment				
	Straw bedding	40	3 (7.5)	0 (0)	0.077
	Total	120	7 (5.8)	0 (0)	0.007
Total prevalence	Animal samples				
	Fecal samples	120	24 (20)	3 (2.5)	≤0.001
	Milk	120	9 (7.5)	0 (0)	0.002
	Total	240	33 (13.7)	3 (1.2)	≤0.001
	Environment				
	Straw bedding	120	15 (12.5)	2 (1.6)	0.001
	Total	360	48 (13.3)	5 (5.3)	≤0.001

**Table 2:** Antibiogram of isolated *L. monocytogenes* strains.

Antibiotics	Sensitive No. (%)	Resistant No. (%)
Imipenem	4/48 (8.3%)	44/48 (91.6%)
Penicillin G	2/48 (4.1%)	46/48 (95.8%)
Erythromycin	29/48 (60.4)	19/48 (39.6%)
Amikacin	25/48 (52.1%)	23/48 (47.9%)
Streptomycin	2/48 (4.1%)	46/48 (95.8%)
Gentamicin	28/48 (58.3%)	20/48 (41.7%)
Vancomycin	47/48 (97.9%)	1/48 (2.1%)
SXT ^1^	0 (0%)	48/48 (100%)
Levofloxacin	40/49 (81.6%)	8/48 (16.6%)

^1^ Sulfamethoxazole–trimethoprim.

**Table 3:** Prevalence and distribution of *L. monocytogenes* virulence genes with their antimicrobial resistance profile and MAR index.

Samples ID	Sample Source	Distribution of Virulence Gene			Antimicrobial Profile	MAR Index
		*iap*	*hylA*	*actA*		
5	Feces	+	+		IMP, Pen G, STM, SXT, E, AK, LEV	0.777
7	Feces	+			IMP, Pen G, STM, SXT, E	0.555
10	Feces	+			IMP, Pen G, STM, SXT, AK	0.555
11	Feces	+			IMP, Pen G, STM, SXT, AK, G	0.666
13	Feces	+			IMP, Pen G, STM, SXT, G	0.555
17	Feces	+			IMP, Pen G, STM, SXT, E, G	0.666
19	Feces	+			IMP, Pen G, STM, SXT, AK	0.555
22	Feces	+	+		IMP, Pen G, STM, SXT, AK, G	0.666
28	Feces	+	+		IMP, Pen G, STM, SXT, AK, G, LEV	0.777
43	Milk	+	+		IMP, Pen G, STM, SXT, E, AK, LEV	0.777
48	Milk	+			IMP, Pen G, STM, SXT, G	0.555
76	Milk	+			IMP, Pen G, STM, SXT, VA, E, G	0.777
78	Milk	+			IMP, Pen G, STM, SXT, E, G	0.666
79	Milk	+	+	+	IMP, Pen G, STM, SXT, E, AK, G, LEV	0.888
89	Bedding	+	+		IMP, Pen G, STM, SXT, AK	0.555
90	Bedding	+			IMP, Pen G, STM, SXT, LEV	0.555
101	Bedding	+			IMP, Pen G, STM, SXT, G	0.555
107	Bedding	+			IMP, Pen G, STM, SXT, E	0.555
110	Bedding	+	+		IMP, Pen G, STM, SXT, AK	0.555
112	Bedding	+	+	+	IMP, Pen G, STM, SXT, E, AK, G	0.777
113	Bedding	+			IMP, Pen G, STM, SXT, G	0.555
Prevalence of virulence genes in Household I no. (%) *p*-value ≤ 0.001 *		*iap* 21 (100)	*hylA* 8 (38)	*actA* 2 (9.5)	Collective MAR index in Household I = 0.644	
9	Feces	+			IMP, Pen G, STM, SXT,	0.444
12	Feces	+		+	IMP, Pen G, STM, SXT, AK	0.555
15	Feces	+			IMP, Pen G, STM, SXT, G, LEV	0.666
20	Feces	+			IMP, Pen G, STM, SXT, E	0.555
23	Feces	+	+		IMP, Pen G, STM, SXT, AK, G	0.666
28	Feces	+	+		IMP, Pen G, STM, SXT, E, AK	0.666
30	Feces	+			IMP, Pen G, STM, SXT, E	0.555
34	Feces	+	+		IMP, Pen G, STM, SXT, AK	0.555
37	Feces	+			IMP, Pen G, STM, SXT	0.444
38	Feces	+			IMP, Pen G, STM, SXT, AK	0.555
40	Feces	+			IMP, Pen G, STM, SXT, G, LEV	0.666
49	Milk	+	+	+	IMP, Pen G, STM, SXT, E, AK	0.666
50	Milk	+	+		IMP, Pen G, STM, SXT, E, AK	0.666
56	Milk	+	+		IMP, Pen G, STM, SXT, AK	0.555
70	Milk	+			IMP, Pen G, STM, SXT, E, G	0.666
87	Bedding	+	+		IMP, Pen G, STM, SXT, AK	0.555
105	Bedding	+			IMP, Pen G, STM, SXT, AK, G	0.666
107	Bedding	+	+	+	IMP, Pen G, STM, SXT, E, AK, G	0.777
111	Bedding	+			IMP, Pen G, STM, SXT, E, G	0.666
112	Bedding	+	+		IMP, Pen G, STM, SXT, AK, G	0.666
Prevalence of virulence genes in Household II no. (%) at *p*-value ≤ 0.001 *		*iap* 20 (100)	*hylA* 9 (45)	*actA* 3 (15)	Collective MAR index in Household II = 0.610	
1	Feces	+			IMP, STM, SXT,	0.333
3	Feces	+	+		Pen G, STM, SXT, E	0.444
5	Feces	+		+	IMP, Pen G, SXT, E	0.444
7	Feces	+		+	STM, SXT, LEV	0.333
90	Bedding	+			IMP, Pen G, SXT,	0.333
91	Bedding	+	+		Pen G, STM, SXT,	0.333
93	Bedding	+			Pen G, STM, SXT,	0.333
Prevalence of virulence genes in Household III no. (%) at *p*-value 0.011 *		*iap* 7 (100)	*hylA* 2 (28.5)	*actA* 2 (28.5)	Collective MAR index in Household III = 0.364	

+: the presence of the gene, *: significance difference, STM: streptomycin, Pen G: penicillin G, SXT: sulfamethoxazole–trimethoprim, IMP: imipenem, E: erythromycin, AK: amikacin, LEV: levofloxacin, VA: vancomycin, G: gentamicin.

**Table 4:** MIC and MMC values of rose water, rose, and orange oil using Tween 80 as an emulsifier for oil. Concentrations of rose water, rose oil, and orange oil are given in mg/L.

Microorganisms	Rose Water (mg/L)		Rose Oil (mg/L)		Orange Oil (mg/L)	
	MIC	MMC	MIC	MMC	MIC	MMC
*L. monocytogenes*	10	10	2.5	20	7.8	7.8
*C. sakazakii*	20	20	20	40	7.8	7.8

**Table 5:** Oligonucleotide sequence for the primers used in the study.

Target Gene	Oligonucleotide Sequence (5′ → 3′)	Virulence Factor	Size (bp)	Reference
*iap* (F)	ACAAGCTGCACCTGTTGCAG	Invasive associated protein	131	[12]
*iap* (R)	TGACAGCGTGTGTAGTAGCA			
*hlyA* (F)	GCAGTTGCAAGCGCTTGGAGTGAA	Hemolysin	456	[12]
hlyA (R)	GCAACGTATCCTCCAGAGTGATCG			
*actA* (F)	CGCCGCGGAAATTAAAAAAAGA	Actin polymerization protein	839	[26]
actA (R)	ACGAAGGAACCGGGCTGCTAG			
*cgcA* (F)	GGCGGACGAAGCCTCAGAGAGT	diguanylate cyclase -Encoding Gene, *cgcA* (species specific)	492	[49]
*cgcA* (R)	TTAGGGCCATTCGGAAATCCGAA			

## Data Availability

The data that support the findings of this study are available within the article.
